# Vasopressin for hypotensive paediatric intensive care patients: an ESPNIC endorsed international survey: the P-PRESS survey

**DOI:** 10.1016/j.aicoj.2026.100168

**Published:** 2026-09-22

**Authors:** Vladimir L. Cousin, Akash Deep, Mathieu Jozwiak, Pierre Tissieres

**Affiliations:** a IHU-SEPSIS Comprehensive Sepsis Center, Paediatric Intensive Care, Neonatal Medicine and Paediatric Emergency Department, AP-HP Paris Saclay University, Bicêtre Hospital, Le Kremlin-Bicêtre, France; b Paediatric Intensive Care Unit, King’s College Hospital NHS Foundation Trust, London, United Kingdom; c Service de Médecine Intensive Réanimation, Centre Hospitalier Universitaire l’Archet 1, Nice, France et UR2CA, Université Côte d’Azur, France; d Institute of Integrative Cell Biology, CNRS, CEA, Paris Saclay University, Gif-sur-Yvette, France

## Abstract

NAD, noradrenalin; MAP, mean arterial pressure; DAP, diastolic arterial pressure; UI, International unit. *Noradrenalin tartrate.

Dear editor,

In severe septic shock, increasing doses of catecholaminergic drugs can lead to side effects, including potential immune suppression and direct cellular toxicities [1]. In this context, the concept of “decatecholaminisation” has emerged, proposing the addition of other vasopressors alongside catecholamines [1]. Vasopressin, by activating V1 receptors, exerts a potent vasopressive effect, significantly reducing noradrenaline doses while maintaining arterial pressure [1]. Despite any impact on patients’ outcomes in adult or paediatric trials, vasopressin use has expanded in Paediatric Intensive Care Units (PICUs) [2]. Furthermore, the 2026 Surviving Sepsis Campaign in Children now recommends adding vasopressin in cases of persistent shock despite high doses of catecholamines [3].

However, no consensus exists regarding the use of vasopressin in clinical settings. To address these gaps, we conducted an international survey endorsed by the European Society of Paediatric and Neonatal Intensive Care (ESPNIC). The complete survey methodology is detailed in the supplementary files and follows CHERRIES (Checklist for Reporting Results of Internet E-Surveys) guidelines for methodology and results reporting. The survey was hosted and electronically distributed as an open survey via the SurveyMonkey platform (SurveyMonkey Inc., California, USA) from 07/2023 to 09/2024. It was distributed electronically to all ESPNIC members and promoted on the ESPNIC website. Additionally, it was promoted on the World Federation of Paediatric and Critical Care Societies website.

A total of 95 respondents completed the survey, representing 21% of 457 registered members, with complete results shown in Supplementary Table S1. Overall, 58% of respondents worked in Europe, and 74% were PICU specialists with more than 5 years of experience. The respondents' first-line vasopressor was noradrenaline in 75% of respondents. Vasopressin was selected as a second-line vasopressor by 44% of respondents and as a third-line vasopressor by 40% of respondents (Supplementary Table S1). The two main indications for vasopressin use were refractory vasoplegic shock with preserved cardiac function (79% of respondents) and septic shock unresponsive to initial resuscitation (66% of respondents). Existing mesenteric or digital ischemia were the main declared contraindications to vasopressin use, and when used, clinicians' main concerns were tissue ischemia (Fig. 1). A noradrenaline dose of 0.5 µg/kg/min for 2−6 hours was the most commonly selected trigger for initiating vasopressin (Fig. 1). Clinical signs or the noradrenaline infusion used as triggers for vasopressin initiation varied widely among respondents (Supplementary figures S1 and S2). Reported initial vasopressin doses ranged from 0.0001 U/kg/min to 0.04 U/min (Fig. 1). Once started, the vast majority of respondents titrated vasopressin based on clinical response (73% of respondents). Regarding the maximum dose, a wide range was reported, from 0.001 U/kg/min to 0.5 U/kg/min, with no maximal dose specified by 15% of respondents (Fig. 1). Vasopressin was reported to be weaned first by 67% of respondents, and 94% indicated a gradual weaning process.

**Fig. 1:**
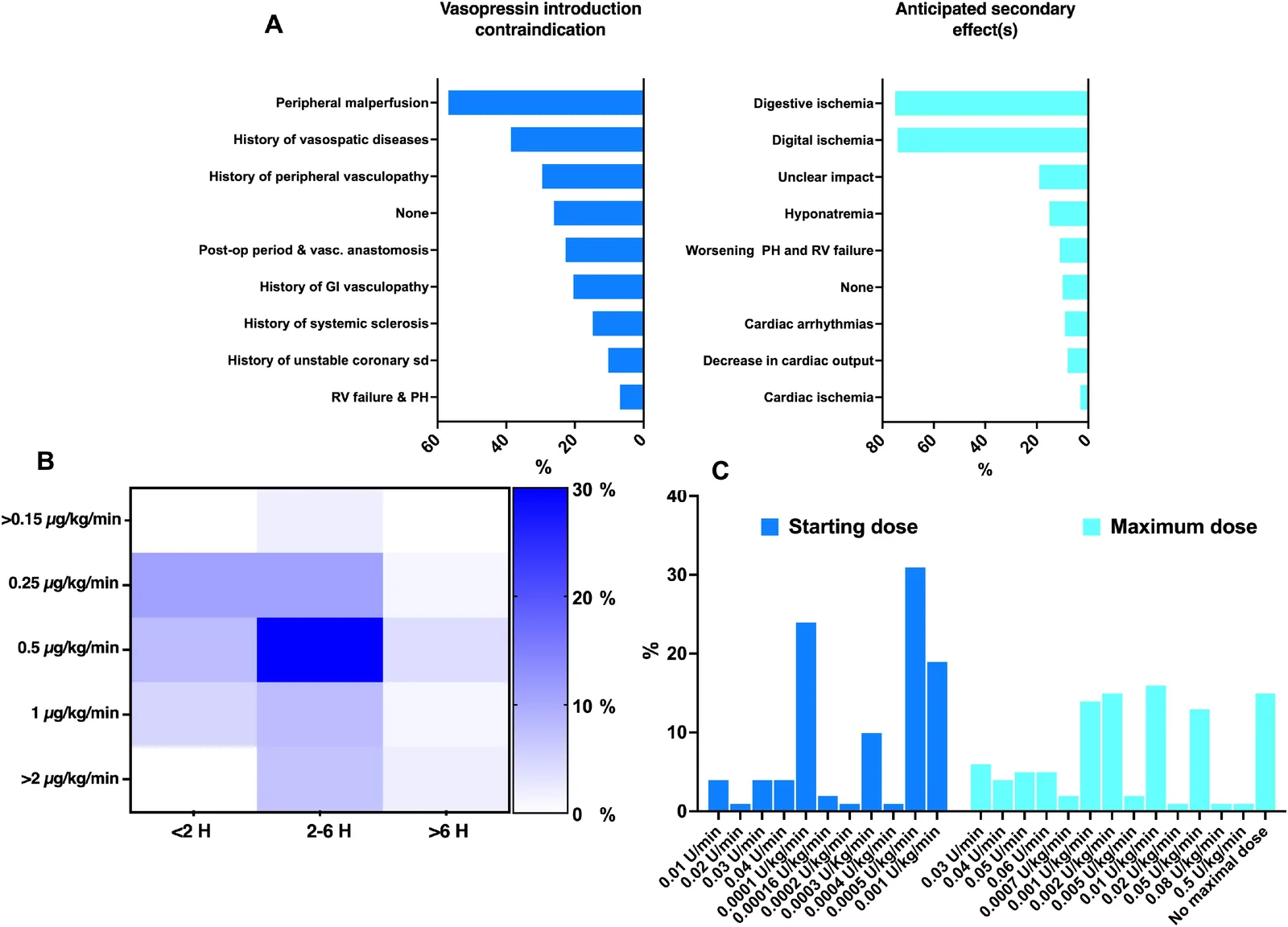
(A) Contraindications and secondary effects anticipated with vasopressin infusion. Percentage (%) are depicted for each variable. GI, gastrointestinal; RV, right ventricle; PH, pulmonary hypertension. (B) Heatmap of noradrenaline dose and duration as a trigger for vasopressin infusion initiation. Heatmap showing the percentage (%) of responders for the combination of both the dose in µg/kg/min and the duration of noradrenaline infusion in hours as a trigger for vasopressin initiation. (C) Vasopressin initial and maximum dose during vasopressin infusion. Bar graphs showing dose management of the vasopressin infusion, with proportion of respondent on the Y axis and different dose in µg/kg/minute on the X axis. The upper panel shows the initial dose in µg/kg/min and the lower panel the maximum dose in mcg/kg/min.

Use of vasopressin by experienced PICU clinicians showed variation in its indications, initiation triggers, dosages, and weaning practices. There is a notable lack of specific guidance on vasopressin use modalities in children, especially the timing of initiation. The wide range of doses and durations of first-line vasopressor infusion that trigger vasopressin initiation, as reported in the survey, reflects the ongoing debate in the literature regarding the timing of vasopressin initiation. In adults, the duration and dose of noradrenaline were reported in the PRESS survey, with a duration <6 h and a dose of 0.25−0,5 µg/kg/min mostly used as a vasopressin initiation trigger [4]. Currently, no definitive cutoff has been defined for vasopressin initiation, but recent literature suggests improved outcomes when vasopressin is commenced at a lower noradrenaline dose or after a shorter duration of noradrenaline support [5,6]. Further studies are needed to better determine the appropriate timing of vasopressin introduction, potentially with better stratification of patients’ hemodynamic profiles and vasopressor kinetics. Once initiated, reported vasopressin dosing varies considerably, particularly for maximal doses, as 15% of respondents do not use a maximal dose of vasopressin in their practice. Such practices stand out because they appear to exceed doses reported in paediatric studies and in both the VANISH and VASST studies, which could be scaled based on weight to doses from 0.0001 to 0.0005 U/kg/min [2,7,8]. Eventually, vasopressors may be weaned, and in the presence of vasopressin and noradrenaline, the optimal methods for weaning remain largely unclear in the current literature [9,10]. In the current survey, most respondents prefer weaning vasopressin before noradrenaline. Vasopressor weaning remains a critical period, and continuous, careful assessment of perfusion is essential during this phase.

This survey has certain limitations, the main one being the small number of respondents, mostly from Europe. The survey was conducted solely in English, which might have excluded non-English speakers from participating. Predefined answers for triggers used for vasopressin initiation, infusion, and weaning may lack granularity and potentially oversimplify those specific points. Finally, there was a lack of details regarding which form of noradrenaline (“base” vs “tartrate”) was used by respondents, as it could impact the doses reported.

This international survey showed that vasopressin is primarily used as a second-line vasopressor, with varied triggers for initiating treatment and different initial and titrated doses of vasopressin. It provides contemporary international insight into reported practices of vasopressin use in children experiencing shock.

## Author contributions

VLC, MJ, PT conceived and designed the survey. VLC, MJ and PT analyzed and interpreted the data. VLC and MJ drafted the first version of the manuscript report. All authors contributed drafting the manuscript and approved the final version of the manuscript.

## Consent for publication

Not applicable.

## Ethics approval and consent to participate

The survey was endorsed by the European Society of Pediatric and Neonatal Intensive care (ESPNIC).

## Funding

No funding to declare.

## Availability of data and materials

The datasets used and/or analysed during the current study are available from the corresponding author on reasonable request.

## Declaration of competing interest

PT received consulting fees from Thermo Fisher, Sedana, Baxter, and Viatris, and is a member of the steering committee of the Surviving Sepsis Campaign and co-chair of the Paediatric guidelines. The other authors have no conflicts of interest to declare
