# Supplementary material for: Vasopressin for hypotensive paediatric intensive care patients: an ESPNIC endorsed international survey: the P-PRESS survey
Source: Ann Intensive Care. 2026 Sep 22;16:100168. doi: 10.1016/j.aicoj.2026.100168 (PMC13673358; doi:10.1016/j.aicoj.2026.100168)
Supplement: Supplementary file 1 [file mmc1.docx]

**SUPPLEMENTARY FILE**

to Cousin VL et al. Vasopressin for hypotensive paediatric intensive care patients: an ESPNIC endorsed international survey: the P-PRESS survey

**Supplementary methods**

**Complete survey’s methodology**

**Survey development:** The survey was designed to evaluate the clinical use of vasopressin. Particular area of interest for this survey were respondent characteristics, reluctance to use vasopressin, initiation of vasopressin (triggers and dosage), and infusion of vasopressin (dose titration and adjustment) and weaning. Questions were independently developed based on uncertainty in current guidelines on vasopressin by paediatric (VLC, PT) and adult (MJ) intensivists [15]. Redundant items were removed, and the remaining questions were assessed collaboratively by all co-authors. A panel of paediatric intensivists evaluated the survey for its feasibility and duration, with adjustments made based on their feedback. The final survey included 19 questions divided in the four subsections of interest.

**Survey dissemination and population:** The survey was hosted and electronically distributed as an open survey via the SurveyMonkey platform (SurveyMonkey Inc., San Mateo, California, USA), in English, from 07/2023 to 09/2024. Responses were collected at the individual intensivist level rather than as a single unit-wide submission, due to the suspected variation in practices regarding vasopressin use among individuals. Once submitted, responses could not be reviewed or amended and respondents received an alert before final submission. No personal data was collected and participation was anonymous with no login required. Participation was only voluntary and no incentive was offered for the completion of the survey. The survey was distributed electronically to every ESPNIC members who were invited via email to fulfil the survey trough the survey link. The survey was promoted on the ESPNIC website while it was open and members received regularly the ESPNIC newsletter which included information regarding the survey and the link for completion as a reminder. Additionally, the survey was promoted on World Federation of Paediatric and Critical Care Societies website and members of these societies were invited to participate via email, which included the survey link.

**Statistical analysis:** Data were summarised and presented through descriptive statistics, with numbers and percentages for categorical variables. Continuous variables are described using the median and interquartile range. No comparative analysis was performed, as the primary aim of this work was descriptive, and no specific subgroups were identified that required targeted comparisons. Data were analysed using Microsoft Excel (MS Excel Microsoft Corporation 2024).

**Supplementary Table 1. Full survey response**

| **Question** | **N** | **%** |
| --- | --- | --- |
| 1. **Location** |  |  |
| Europe | 55 | 58 |
| North america | 11 | 11 |
| South america | 14 | 15 |
| Asia/Pacific | 14 | 15 |
| Africa | 1 | 1 |
| **2. Experience as intensivist** |  |  |
| Not specialized yet, in training | 8 | 8 |
| <2 y | 4 | 4 |
| 2-5y | 13 | 14 |
| >5y | 70 | 74 |
| **3. Type of institution** |  |  |
| University Hospital | 77 | 81 |
| Non-university public hospital | 10 | 11 |
| Private Hospital | 7 | 7 |
| other | 1 | 1 |
| **4. Number of ICU beds** |  |  |
| <5 | 4 | 4 |
| 5-10 | 35 | 37 |
| 11-15 | 23 | 24 |
| 16-20 | 16 | 17 |
| >20 | 17 | 18 |
| **5. Number of patients admitted per year for septic shock** |  |  |
| <500 | 67 | 71 |
| 500-1000 | 16 | 17 |
| 1000-1500 | 8 | 8 |
| 1500-2000 | 2 | 2 |
| >2000 | 2 | 2 |
| **6. In which situation would you not introduce vasopressin? *** |  |  |
| None | 23 | 26 |
| History of GI vasculopathy | 18 | 20 |
| History of systemic sclerosis | 13 | 14 |
| History of vasospatic diseases (Raynaud disease, acrocyanosis, livedo reticularis) | 34 | 38 |
| History of peripheral vasculopathy | 26 | 29 |
| Peripheral malperfusion already present (digital ischemia, non-occlusive mesenteric ischemia) | 50 | 56 |
| Postoperative period with vascular anastomosis | 20 | 22 |
| Right ventricular failure with severe pulmonary hypertension | 6 | 6 |
| History of unstable coronary syndrome | 9 | 10 |
| *No response* | *7* |  |
| **7. Feared secondary effects while using vasopressin? *** |  |  |
| Digital ischemia | 66 | 74 |
| Digestive ischemia | 67 | 75 |
| Unclear impact on outcome | 17 | 19 |
| Hyponatremia | 14 | 15 |
| Cardiac arrhythmias | 9 | 9 |
| Cardiac ischemia | 3 | 3 |
| Decrease in cardiac output | 8 | 8 |
| Worsening of existing severe pulmonary hypertension and right ventricular failure | 10 | 11 |
| None | 9 | 10 |
| *No response* | *6* |  |
| **8. In which situations you would mostly use vasopressin? *** | | |
| Septic shock refractory to initial resuscitation | 59 | 66 |
| Septic shock with high risk of renal failure | 7 | 7 |
| Septic shock in a patient with ARDS | 14 | 15 |
| Septic shock in a cirrhotic patient | 20 | 22 |
| Septic shock with atrial arrhythmia | 12 | 13 |
| Refractory vasoplegic shock (other than septic) without reduced cardiac function | 71 | 79 |
| Refractory vasoplegic shock (other than septic) with reduced cardiac function | 30 | 33 |
| Refractory vasoplegic shock (other than septic) with right ventricular dysfunction | 10 | 11 |
| Refractory shock (any type) as a catecholamine sparing agent | 17 | 19 |
| *No response* | *6* |  |
| **9. At which step of resuscitation do you start vasopressin?** |  |  |
| First line vasopressor | 0 | 0 |
| Second line, after initial resuscitation using fluid and norepinephrine | 42 | 44 |
| Third line, as rescue therapy | 38 | 40 |
| Early in the vasopressive therapy to reach pressure goal and spare catecholamine in order to decrease catecholamine load | 6 | 6 |
| other / no answer | 9 | 10 |
|  |  |  |
| **10. What is you first line vasopressor?** |  |  |
| Norepinephrine | 65 | 75 |
| Epinephrine | 20 | 23 |
| Vasopressin | 0 | 0 |
| Angiotensin II | 0 | 0 |
| Phenylephrine | 1 | 1 |
| Methylene blue | 0 | 0 |
| Dopamine | 1 | 1 |
| *No response* | *8* |  |
| **11. If you have to choose a norepineprine dose as a trigger to initiate vasopressin, what dose would it be (Noradrenaline equivalent dose µg/kg/min)?** |  |  |
| none | 3 | 3 |
| 0,15-0,25 µg/kg/min | 6 | 7 |
| 0.25-0.5 µg/kg/min | 32 | 37 |
| 0.5-1 µg/kg/min | 32 | 37 |
| 1-2 µg/kg/min | 8 | 9 |
| 2-5 µg/kg/min | 6 | 7 |
| No response | 8 |  |
| **12. If you have to choose a duration of noradrenaline support as a trigger to initiate vasopressin, what duration would it be (hours of support)?** |  |  |
| none | 11 | 13 |
| <2h | 15 | 17 |
| 2-4h | 23 | 26 |
| 2-6h | 8 | 9 |
| 4-6h | 14 | 16 |
| 6-8h | 4 | 5 |
| 6-12h | 1 | 1 |
| 8-12h | 0 | 0 |
| >12h | 11 | 13 |
| *No response* | *8* |  |
| **13. If you have to choose the association of dose and duration of noradrenaline support as a trigger to initiate vasopressin, what would it be (Noradrenaline equivalent dose µg/kg/min and duration of support)?** |  |  |
| >0.15 µg/kg/min for 2-6 hours | 2 | 2 |
| 0.25 µg/kg/min for <2 hours | 9 | 11 |
| 0.5 µg/kg/min for <2 hours | 7 | 8 |
| 1 for <2 hours | 4 | 5 |
| >2 for <2 hours | 0 | 0 |
| 0.25 for 2-6 hours | 9 | 11 |
| 0.5 for 2-6 hours | 26 | 30 |
| 1 for 2-6 hours | 7 | 8 |
| >2 for 2-6 hours | 5 | 6 |
| 0.25 for >6 hours | 1 | 1 |
| 0.5 for >6 hours | 3 | 4 |
| 1 for >6hours | 2 | 2 |
| >2 for >6 hours | 2 | 2 |
| >2 µg/kg/min for 2-6 hours | 1 | 1 |
| Duration is not a trigger | 8 | 9 |
| *No response* | *9* |  |
| **14. If you have to choose a clinical sign as a trigger to initiate vasopressin, what would it be? *** |  |  |
| Persistent skin mottling | 7 | 8 |
| Oliguria | 12 | 14 |
| Persistent hyperlactatemia | 11 | 12 |
| Persistent low systolic arterial pressure | 14 | 16 |
| Persistent low mean arterial pressure | 55 | 63 |
| Persistent low diastolic arterial pressure | 31 | 36 |
| *No response* | *8* |  |
| **15. What vasopressin starting dose do you use?** |  |  |
| 0.01 UI/min | 3 | 4 |
| 0,02 UI/min | 1 | 1 |
| 0,03 Ui/min | 3 | 4 |
| 0.04 UI/min | 3 | 4 |
| 0.0001 UI/kg/min | 19 | 24 |
| 0,00016 Ui/kg/min | 2 | 2 |
| 0,0002 Ui/kg/min | 1 | 1 |
| 0,0003 UI/Kg/min | 8 | 10 |
| 0,0004 Ui/kg/min | 1 | 1 |
| 0.0005 UI/kg/min | 25 | 31 |
| 0.001 UI/kg/min | 15 | 19 |
| *No response* | *12* |  |
| **16. What vasopressin dose do you use during the patient’s resuscitation?** |  |  |
| Other (specify in next question) | 3 | 4 |
| Fixed dose same as initial dose | 12 | 15 |
| Fixed dose at 0.01 UI/min | 2 | 2 |
| Fixed dose at 0.02 UI/min | 1 | 1 |
| Fixed dose at 0.03 UI/min | 1 | 1 |
| Fixed dose at 0.05 UI/min | 1 | 1 |
| Fixed dose at 0.06 UI/min | 0 | 0 |
| Fixed dose at 0.0001 UI/kg/min | 2 | 2 |
| Fixed dose at 0.01 UI/kg/min | 1 | 1 |
| Titration to clinical response | 60 | 73 |
| *No response* | *12* |  |
| **17. What is the maximal vasopressin dose you may use?** |  |  |
| 0,03 UI/min | 5 | 6 |
| 0,04 Ui/min | 3 | 4 |
| 0,05 UI/min | 4 | 5 |
| 0,06 UI/min | 4 | 5 |
| 0,0007 Ui/kg/min | 2 | 2 |
| 0,001 UI/kg/min | 11 | 14 |
| 0,002 UI/kg/min | 12 | 15 |
| 0,005 Ui/kg/min | 2 | 2 |
| 0,01 UI/kg/min | 13 | 16 |
| 0,02 Ui/kg/min | 1 | 1 |
| 0,05 UI/kg/min | 10 | 13 |
| 0,08 UI/kg/min | 1 | 1 |
| 0,5 Ui/kg/min | 1 | 1 |
| No maximal dose | 12 | 15 |
| *No response* | *12* |  |
| **18. How do you manage the cessation of vasopressin support with another vasopressor?** |  |  |
| I stopped vasopressin in parallel to another vasopressor | 16 | 20 |
| I stopped vasopressin after norepinephrine or first line vasopressor regardless of the vasopressor use | 11 | 13 |
| I stopped vasopressin first | 55 | 67 |
| *No response* | *13* |  |
| **19. How do you stop vasopressin support?** |  |  |
| Progressive decreasing of the vasopressin support | 78 | 94 |
| Abrupt cessation of the vasopressin support | 5 | 6 |
| *No response* | *12* |  |

* mulitples answers were possible

**Supplementary Figure 1**

Dose and duration of noradrenaline used as a trigger for vasopressin initiation

Upper panel: Dose of noradrenaline used as a trigger for vasopressin initiation

Lower panel: Duration of noradrenaline used as a trigger for vasopressin initiation
